# Supplementary material for: A Critical View of the Application of the APEX Software (Aqueous Photochemistry of Environmentally-Occurring Xenobiotics) to Predict Photoreaction Kinetics in Surface Freshwaters
Source: Molecules. 2019 Dec 18;25(1):9. doi: 10.3390/molecules25010009 (PMC7017383; doi:10.3390/molecules25010009)
Supplement: Supplementary file 1 [file molecules-25-00009-s001.zip › 3.2.4_gcc-4.4.0.zip!/3.2.4_gcc-4.4.0/3.2.4_gcc-4.4.0/doc/octave/html/liboctave/Matrix-Factorizations.html]

Matrix Factorizations - Octave C++ Classes


Next: Ranges,
Previous: Matrix and Vector Operations,
Up: Top


---

## 5 Matrix Factorizations

— : **AEPBALANCE** (void)  
— : **AEPBALANCE** (const Matrix &a, const char \*balance\_job)  
— : **AEPBALANCE** (const AEPBALANCE &a)

— : AEPBALANCE& **operator =** (const AEPBALANCE &a)

— : Matrix **balanced\_matrix** (void) const  
— : Matrix **balancing\_matrix** (void) const

— : ostream& **operator <<** (ostream &os, const AEPBALANCE &a)

— : **ComplexAEPBALANCE** (void)  
— : **ComplexAEPBALANCE** (const ComplexMatrix &a, const char \*balance\_job)  
— : **ComplexAEPBALANCE** (const ComplexAEPBALANCE &a)

— : ComplexAEPBALANCE& **operator =** (const ComplexAEPBALANCE &a)

— : ComplexMatrix **balanced\_matrix** (void) const  
— : ComplexMatrix **balancing\_matrix** (void) const

— : ostream& **operator <<** (ostream &os, const ComplexAEPBALANCE &a)

— : **DET** (void)  
— : **DET** (const DET &a)

— : DET& **operator =** (const DET &a)

— : int **value\_will\_overflow** (void) const  
— : int **value\_will\_underflow** (void) const

— : double **coefficient** (void) const  
— : int **exponent** (void) const  
— : double **value** (void) const

— : ostream& **operator <<** (ostream &os, const DET &a)

— : **ComplexDET** (void)  
— : **ComplexDET** (const ComplexDET &a)

— : ComplexDET& **operator =** (const ComplexDET &a)

— : int **value\_will\_overflow** (void) const  
— : int **value\_will\_underflow** (void) const

— : Complex **coefficient** (void) const  
— : int **exponent** (void) const  
— : Complex **value** (void) const

— : ostream& **operator <<** (ostream &os, const ComplexDET &a)

— : **GEPBALANCE** (void)  
— : **GEPBALANCE** (const Matrix &a, const Matrix &, const char \*balance\_job)  
— : **GEPBALANCE** (const GEPBALANCE &a)

— : GEPBALANCE& **operator =** (const GEPBALANCE &a)

— : Matrix **balanced\_a\_matrix** (void) const  
— : Matrix **balanced\_b\_matrix** (void) const  
— : Matrix **left\_balancing\_matrix** (void) const  
— : Matrix **right\_balancing\_matrix** (void) const

— : ostream& **operator <<** (ostream &os, const GEPBALANCE &a)

— : **CHOL** (void)  
— : **CHOL** (const Matrix &a)  
— : **CHOL** (const Matrix &a, int &info)  
— : **CHOL** (const CHOL &a)

— : CHOL& **operator =** (const CHOL &a)

— : Matrix **chol\_matrix** (void) const

— : ostream& **operator <<** (ostream &os, const CHOL &a)

— : **ComplexCHOL** (void)  
— : **ComplexCHOL** (const ComplexMatrix &a)  
— : **ComplexCHOL** (const ComplexMatrix &a, int &info)  
— : **ComplexCHOL** (const ComplexCHOL &a)

— : ComplexCHOL& **operator =** (const ComplexCHOL &a)

— : ComplexMatrix **chol\_matrix** (void) const

— : ostream& **operator <<** (ostream &os, const ComplexCHOL &a)

— : **HESS** (void)  
— : **HESS** (const Matrix &a)  
— : **HESS** (const Matrix&a, int &info)  
— : **HESS** (const HESS &a)

— : HESS& **operator =** (const HESS &a)

— : Matrix **hess\_matrix** (void) const  
— : Matrix **unitary\_hess\_matrix** (void) const

— : ostream& **operator <<** (ostream &os, const HESS &a)

— : **ComplexHESS** (void)  
— : **ComplexHESS** (const ComplexMatrix &a)  
— : **ComplexHESS** (const ComplexMatrix &a, int &info)  
— : **ComplexHESS** (const ComplexHESS &a)

— : ComplexHESS& **operator =** (const ComplexHESS &a)

— : ComplexMatrix **hess\_matrix** (void) const  
— : ComplexMatrix **unitary\_hess\_matrix** (void) const

— : ostream& **operator <<** (ostream &os, const ComplexHESS &a)

— : **SCHUR** (void)  
— : **SCHUR** (const Matrix &a, const char \*ord)  
— : **SCHUR** (const Matrix &a, const char \*ord, int &info)  
— : **SCHUR** (const SCHUR &a, const char \*ord)

— : SCHUR& **operator =** (const SCHUR &a)

— : Matrix **schur\_matrix** (void) const  
— : Matrix **unitary\_matrix** (void) const

— : ostream& **operator <<** (ostream &os, const SCHUR &a)

— : **ComplexSCHUR** (void)  
— : **ComplexSCHUR** (const ComplexMatrix &a, const char \*ord)  
— : **ComplexSCHUR** (const ComplexMatrix &a, const char \*ord, int &info)  
— : **ComplexSCHUR** (const ComplexSCHUR &a, const char \*ord)

— : ComplexSCHUR& **operator =** (const ComplexSCHUR &a)

— : ComplexMatrix **schur\_matrix** (void) const  
— : ComplexMatrix **unitary\_matrix** (void) const

— : ostream& **operator <<** (ostream &os, const ComplexSCHUR &a)

— : **SVD** (void)  
— : **SVD** (const Matrix &a)  
— : **SVD** (const Matrix &a, int &info)  
— : **SVD** (const SVD &a)

— : SVD& **operator =** (const SVD &a)

— : DiagMatrix **singular\_values** (void) const  
— : Matrix **left\_singular\_matrix** (void) const  
— : Matrix **right\_singular\_matrix** (void) const

— : ostream& **operator <<** (ostream &os, const SVD &a)

— : **ComplexSVD** (void)  
— : **ComplexSVD** (const ComplexMatrix &a)  
— : **ComplexSVD** (const ComplexMatrix &a, int &info)  
— : **ComplexSVD** (const ComplexSVD &a)

— : ComplexSVD& **operator =** (const ComplexSVD &a)

— : DiagMatrix **singular\_values** (void) const  
— : ComplexMatrix **left\_singular\_matrix** (void) const  
— : ComplexMatrix **right\_singular\_matrix** (void) const

— : ostream& **operator <<** (ostream &os, const ComplexSVD &a)

— : **EIG** (void)  
— : **EIG** (const Matrix &a)  
— : **EIG** (const Matrix &a, int &info)  
— : **EIG** (const ComplexMatrix &a)  
— : **EIG** (const ComplexMatrix &a, int &info)  
— : **EIG** (const EIG &a)

— : EIG& **operator =** (const EIG &a)

— : ComplexColumnVector **eigenvalues** (void) const

— : ComplexMatrix **eigenvectors** (void) const

— : ostream& **operator <<** (ostream &os, const EIG &a)

— : **LU** (void)  
— : **LU** (const Matrix &a)  
— : **LU** (const LU &a)

— : LU& **operator =** (const LU &a)

— : Matrix **L** (void) const  
— : Matrix **U** (void) const  
— : Matrix **P** (void) const

— : ostream& **operator <<** (ostream &os, const LU &a)

— : **ComplexLU** (void)  
— : **ComplexLU** (const ComplexMatrix &a)  
— : **ComplexLU** (const ComplexLU &a)

— : ComplexLU& **operator =** (const ComplexLU &a)

— : ComplexMatrix **L** (void) const  
— : ComplexMatrix **U** (void) const  
— : Matrix **P** (void) const

— : ostream& **operator <<** (ostream &os, const ComplexLU &a)

— : **QR** (void)  
— : **QR** (const Matrix &A)  
— : **QR** (const QR &a)

— : QR& **operator =** (const QR &a)

— : Matrix **Q** (void) const  
— : Matrix **R** (void) const

— : ostream& **operator <<** (ostream &os, const QR &a)

— : **ComplexQR** (void)  
— : **ComplexQR** (const ComplexMatrix &A)  
— : **ComplexQR** (const ComplexQR &a)

— : ComplexQR& **operator =** (const ComplexQR &a)

— : ComplexMatrix **Q** (void) const  
— : ComplexMatrix **R** (void) const

— : ostream& **operator <<** (ostream &os, const ComplexQR &a)
